# Synthetic Red Blood Cell-Specific Glycolytic Intermediate 2,3-Diphosphoglycerate (2,3-DPG) Inhibits *Plasmodium falciparum* Development *In Vitro*

**DOI:** 10.3389/fcimb.2022.840968

**Published:** 2022-03-15

**Authors:** Inês Morais, Márcia M. Medeiros, Maria Carvalho, Judit Morello, Sara M. Teixeira, Suelma Maciel, Janice Nhantumbo, Ana Balau, Margarida T. G. Rosa, Fátima Nogueira, João Alexandre Rodrigues, Filomena A. Carvalho, Alexandra M. M. Antunes, Ana Paula Arez

**Affiliations:** ^1^ Global Health and Tropical Medicine (GHTM), Instituto de Higiene e Medicina Tropical (IHMT), Universidade NOVA de Lisboa (UNL), Lisbon, Portugal; ^2^ CEDOC, NOVA Medical School, Universidade NOVA de Lisboa, Lisbon, Portugal; ^3^ Centro de Química Estrutural, Instituto Superior Técnico, Universidade de Lisboa, Lisbon, Portugal; ^4^ Clarify Analytical, Évora, Portugal; ^5^ Instituto de Medicina Molecular, Faculdade de Medicina, Universidade de Lisboa, Lisbon, Portugal

**Keywords:** malaria, host–parasite interactions, red blood cell, 2,3-BPG, 2,3-DPG, glycolysis, pyruvate kinase deficiency

## Abstract

Mechanisms of malaria parasite interaction with its host red blood cell may provide potential targets for new antimalarial approaches. Pyruvate kinase deficiency has been associated with resistance to malaria in both experimental models and population studies. Two of the major pyruvate kinase deficient-cell disorders are the decrease in ATP and the increase in 2,3-biphosphoglycerate (2,3-BPG) concentration. High levels of this metabolite, only present in mammalian red blood cell, has an inhibitory effect on glycolysis and we hypothesized that its accumulation may also be harmful to the parasite and be involved in the mechanism of protection provided by that enzymopathy. We examined the effect of a synthetic form, 2,3-DPG, on the *Plasmodium falciparum* intraerythrocytic developmental cycle *in vitro*. Results showed an impairment of parasite growth with a direct effect on parasite maturation as significant lower progeny emerged from parasites that were submitted to 2,3-DPG. Further, adding the compound to the culture medium did not result in any effect on the host cell, but instead the metabolic profile of an infected cell became closer to that of a non-infected cell.

## 1 Introduction

Nowadays, together with COVID-19, malaria is a major public and global health problem, which accounted for 241 million cases and 627 000 deaths in 2020 (World Health Organization, 2021). A significant progress has been made in reducing the malaria burden but a stagnation in the reduction rate has been observed in most recent years in the most historically high-burden malaria countries. Further, this effect is now intensified by a high risk of 19 000 additional deaths in sub-Saharan Africa due to disruptions of national control programs associated with COVID-19 pandemics (World Health Organization, 2020).

Malaria has been one of the strongest selective forces on the human genome, selecting host variants and cellular mechanisms that are determinant for parasite survival, influence pathogenesis and may protect against infection or disease severity (Kariuki and Williams, 2020). The understanding of these mechanisms of parasite dependency on host factors mainly related to the red blood cell (RBC), as well as differences between the biochemistry of host and parasite metabolic pathways may provide potential targets for new approaches.

Pyruvate kinase deficiency (PKD), the most frequent inherited abnormality in RBC glycolysis, is caused by loss-of-function mutations in the pklr gene leading to hemolytic anemia. PKD was associated with resistance to malaria (Min-Oo et al., 2003; Laroque et al., 2017) and babesiosis (Oka et al., 2008) in rodent models and in *in vitro* cultures of *P. falciparum* using human PK-deficient RBC (Ayi et al., 2008; Durand and Coetzer, 2008). This association has been corroborated by population studies (Alves et al., 2010; Machado et al., 2010; Machado et al., 2012). Ayi et al. (2008) suggested that the protective mechanism provided by this erythrocyte enzymopathy may involve a reduction of RBC invasion in case of homozygous subjects and a higher macrophage clearance of ring stage infected RBCs for both heterozygous and homozygous individuals. However, the precise biological and biochemical mechanisms underlying the putative protection of PKD are still unknown.

Pyruvate kinase (PK) catalyzes the conversion of phosphoenolpyruvate to pyruvate, transferring the phosphate group from phosphoenolpyruvate to an ADP molecule producing ATP. Three major cell abnormalities due to PKD are the decrease in ATP and pyruvate production and an increase in 2,3-biphosphoglycerate (2,3-BPG) concentration (Wijk and van Solinge, 2005).

2,3-BPG, the master allosteric regulator of oxygen affinity to hemoglobin, is synthesized on the Rapoport-Luebering shunt, only present in mammalian RBC, by the multifunctional enzyme biphosphoglycerate mutase (BPGM). The increase in 2,3-BPG synthesis found in PKD results in the decrease in ATP synthesis, since an higher activity of the Rapoport-Luebering shunt bypasses the synthesis of ATP catalyzed by phosphoglycerate kinase (PGK) (Wijk and van Solinge, 2005). Also, 2,3-BPG is linked to the conversion of NADH to NAD^+^ by glyceraldehyde-3-phosphate dehydrogenase and in high levels has an inhibitory effect on the glycolytic pathway upstream, precisely the phosphorylation of glucose by hexokinase (Min-Oo and Gros, 2005), and on another metabolic pathway, the pentose phosphate shunt (PPP) (Tomoda et al., 1983).

The metabolic derangements resulting from low intracellular ATP and pyruvate levels lead to the dysfunction of Na+/K+ pump in RBC membrane, inability to maintain the electrochemical gradient and membrane integrity, and inefficient methemoglobin reduction. The failure on restoring two molecules of NAD^+^ upstream, and accumulation of 2,3-BPG may create an unsuitable intracellular environment for parasite development and may be involved in the mechanism of protection against *Plasmodium* infection. Ayi et al. (2009) mimicked ATP depletion phenotype of PKD targeting the RBC enolase, a glycolytic enzyme upstream to PK step, and achieved protection against RBC invasion by *P. falciparum in vitro*. However, a potential role for 2,3-BPG in that protection, which potentially also had increased due to enolase inhibition, was not addressed, and remains unsolved. Further, *Plasmodium falciparum* does not possess BPGM and does not synthesize 2,3-BPG, thus we conjecture that not only this metabolite does not play a role in parasite survival, but on the contrary, it may even be harmful to it.

Here, we examined the effect of 2,3-BPG on the *P. falciparum* intraerythrocytic developmental cycle. To this end, we used 2,3 diphosphoglycerate (2,3-DPG), a synthetic form of 2,3-BPG, used in hematology and transfusion medicine as a reference compound in analysis of RBC glycolytic cycle metabolites, to test whether concentrations of 2,3-BPG above the physiological range inhibits *P. falciparum* development *in vitro*. Next, we compared the inhibitory effect of 2,3-DPG with that of ATP depletion with an enolase inhibitor (sodium fluoride, NaF), which has been previously used to emulate PKD *in vitro*. Finally, we compared the metabolic profiles of infected and non-infected RBCs treated versus not treated with 2,3-DPG.

## 2 Materials and Methods

### 2.1 Blood Donors

Healthy RBCs (type 0) were obtained from adult volunteer donors. Total venous blood was collected into EDTA tubes and immediately washed four times with PBS pH 7.0 to remove plasma and buffy coat. RBCs were left at 50% hematocrit in complete RPMI culture medium [cRPMI, (1.044% (w/v) RPMI 1640 medium (RPMI) (Biowest) supplemented with 0.59% (w/v) HEPES (Sigma-Aldrich), 0.005% (w/v) hypoxanthine (Sigma-Aldrich), 0.5% (w/v) AlbuMAXII (Gibco, Thermo Fisher Scientific) and 0.2% (w/v) of sodium bicarbonate (Merck), buffered to pH 7.0 to 7.2] and stored at 4°C no longer than three weeks to assure no major changes of ATP and 2,3-BPG levels (Capps and Jensen, 1983).

To rule out blood variants that could bias the effect on parasite growth, an aliquot of whole blood was used to perform molecular diagnosis of the most common polymorphisms in Portugal of HBB [A>T mutation on codon 6, exon 1, chr 11 (Waterfall and Cobb, 2001)], pklr [1456C>T mutation on exon 11, chr 1 (Manco and Abade, 2001)] and g6pd [Med mutation, 563C>T mutation on exon 6, chr X, (Tishkoff et al., 2001)] genes.

### 2.2 *Plasmodium falciparum In Vitro* Cultures

Chloroquine (CQ)-sensitive 3D7 *P. falciparum* parasites (BEI Resources MRA-102) were cultivated at standard conditions (5% hematocrit, at 37°C in wet atmosphere with 5% CO2), with daily cRPMI changes according to an adapted protocol from the one developed by Trager and Jensen (1976). Parasite growth was monitored daily by estimating parasite density (percentage of infected-RBCs/total RBCs) in 20% Giemsa-stained (Giemsa′s azur-eosin-methylene blue, Sigma-Aldrich) thin blood smears.

### 2.3 Effect of High Concentrations of 2,3-DPG on Human Cells

According to the oxygen-hemoglobin dissociation curve (Bohr’s effect), that describes the shift in the oxygen dissociation curve to hemoglobin due to changes in the concentration of carbon dioxide, environmental pH and 2,3-BPG concentrations (Hamilton et al., 2004), the physiological concentration range of 2,3-BPG in healthy RBCs varies between 3.6 and 5mM and may increase until 8mM at high altitudes. Based on this range, prior to assays with parasites we tested the potential toxicity for the human cells, of concentrations of synthetic 2,3-Diphospho-D-glyceric acid pentasodium salt (2,3-DPG, Sigma-Aldrich) above the physiological range of the endogenous metabolite. Healthy RBCs and human hepatocellular carcinoma cells [HEPG2 cells (ATCC^®^ HB-8065™)] were used.

#### 2.3.1 Red Blood Cells

RBCs were incubated in triplicate in a 96 well U-bottom microplate for 0, 30, 60 and 90 minutes with 2,3-DPG in 0.9% NaCl according to a two-fold serial dilution (ranging from 16 to 0.25mM), following an adapted protocol from de Freitas et al. (2008) and Evans et al. (2013). To exclude possible confounders due to 2,3-DPG addition such as increase in sodium concentration or changes in pH value, solutions of 1.36% and 0.9% (w/v) NaCl (corresponding to sodium concentrations found in the final concentrations of 16mM and 0.25mM 2,3-DPG solutions, respectively), and solutions of 0.9% NaCl in 0.05M Tris Buffer Saline (TBS) (pH 6.5 and 8.5, corresponding to the pH of the 16mM and 0.25mM solutions, respectively) were used as negative controls. A 20% of Triton X-100 solution in 0.9% NaCl was used as a positive control for RBC lysis. After centrifugation, supernatant was recovered, and absorbance (Abs) was measured at a 450nm wavelength in a luminometer plate reader (Triad TM Series Multimode Detector Dynex Technologies). The percentage of hemolysis was calculated as follows: % Hemolysis= (Sample Abs – Negative Control Abs)/(Positive Control Abs – Negative Control Abs)×100.

#### 2.3.2 HEPG2 Cells

HEPG2 cells were cultivated in T75 culture flasks in cell culture medium [RPMI 1640 medium + GlutaMAX™ (Gibco)], supplemented with 10% (v/v) fetal bovine serum (Sigma-Aldrich) and 1% (v/v) Penicillin/Streptomycin (Sigma-Aldrich) until reaching a confluence of ≥ 75%. After that, HEPG2 cells were detached by trypsinization using Trypsin (Sigma-Aldrich) and Hank’s Balanced Salt solution (HBSS without Ca^2+^, Mg^2+^, Sigma-Aldrich), according to manufacturer’s orientations. Then, 1x10^5^ cells/well were incubated in triplicate in a 96 well flat-bottom microplate with the two-fold serial dilution of 2,3-DPG previously described added to the cell culture medium, for 48 hours. Untreated HEPG2 cells were used as negative controls. Cell viability was assessed after addition of 3-(4,5-Dimethyl-2-thiazolyl)-2,5-diphenyl-2H-tetrazolium bromide, MTT, Methylthiazolyldiphenyl-tetrazolium bromide (M2128 Sigma-Aldrich), following the protocol described in Santos et al. (2015). MTT in culture medium was also added in triplicate to cell-free wells (blank). Absorbance was measured at a 595nm wavelength in the same luminometer plate reader as above. The percentage of cell viability in relation to untreated cells were calculated as follows: % Cell Viability = [(Sample Abs - Blank Abs)/(Control Abs - Blank Abs)] × 100. Absorbance greater than the control indicates cell proliferation and absorbance below the control indicates cell death or inhibition of proliferation. A dose-response analysis was performed to determine the half maximal inhibitory concentration (IC50) of 2,3-DPG against HEPG2 cells in three independent experiments.

### 2.4 Effect of High Concentrations of 2,3-DPG on Malaria Parasite Development

#### 2.4.1 Dose Response Analysis

Prior to all assays, parasite cultures were synchronized by sorbitol treatment with a 5% (w/v) D-sorbitol solution (Sigma-Aldrich) (Radfar et al., 2009) to increase the proportion of ring stages up to 12 hours after invasion.

Based on the physiological range of 2,3-BPG concentration in RBCs, a dose-response analysis was performed to determine the half maximal inhibitory concentration (IC50) of 2,3-DPG against 3D7 *P. falciparum* parasite. Solutions of 2,3-DPG 1.33M were prepared in ultrapure water, from which intermediate dilutions were done with cRPMI. RBCs *ex-vivo* infected initially with synchronized parasite forms at ring stage were put in triplicate in a 96-well flat-bottom microplate culture with cRPMI adjusted to 3% hematocrit and 1% parasite density (at hour 0) in the presence of the two-fold serial dilution of 2,3-DPG (16 – 0.25mM). Infected RBCs incubated without the compound were used as control. SYBR Green (Thermo Fisher Scientific) (0.001% v/v in PBS) was added to each well prepared for each time point (12, 24, 36, 48, 60, 72, 84 and 96 hours of incubation), and plates were incubated again for 1 hour under standard culture conditions. After centrifugation, supernatant was discarded, and cells were resuspended in 1χ PBS. Fluorescence (Relative Fluorescence Units, RFU) was measured on the fluorimeter plate reader (excitation 485nm and emission 535nm). IC50 was determined by plotting parasite density against RFU and analyzed by nonlinear regression (log[inhibitor] vs normalized response) using GraphPad Prism version 8 for Windows (GraphPad Software) (Machado et al., 2016).

#### 2.4.2 Antimalarial Activity of 2,3-DPG

Three biological replicates of synchronized *P. falciparum* cultures maintained as described above, were allowed to grow for one cycle (48h) under daily changed culture media with or without the addition of 2,3-DPG at 8mM (at 0h and 24h). Thin blood smears were daily made to monitor the progress of parasite density (the experiment was repeated three times in a total of eighteen cultures).

Further, during the assays, thin blood smears were daily made to monitor the progress of cultures submitted to different conditions and each blood smear was observed by light microscopy for mature schizonts (at segmented stage), in which it was possible to clearly count the number of resulting merozoites from nuclear division. In total, 10 microscopy fields with approximately 200 cells each in each of 6 smears, from infected treated and untreated RBCs, were blindly examined by two researchers.

#### 2.4.3 Invasion and Maturation Assay and Measurement of ATP Content

To test the effect of 2,3-DPG supplementation on invasion and maturation capacity of the parasite, we kept synchronized cultures with 0.5% of schizont density in 5% hematocrit under standard conditions for two growth cycles (96h). Giemsa-stained thin blood smears were daily prepared to determine the densities of early parasite forms (ring) and of mature parasite forms (late trophozoite plus schizont). RBCs invasion and parasite development inside RBCs, i.e. maturation, were assessed at 24 and 72 hours, and at 48 and 96 hours, respectively.

To exclude a possible 2,3-DPG confounder effect on parasite invasion and/or maturation, such as primary 2,3-DPG effect on RBC ATP content, RBCs were incubated with 8mM 2,3-DPG for 24 hours and then put on *in vitro* culture conditions. ATP content and invasion and maturation levels were compared with the daily addition of 2,3-DPG to culture medium of infected and non-infected RBCs (iRBC and niRBC, respectively).

The ATP depleted phenotype of PKD was induced by blocking RBC glycolytic enzyme enolase (Zerez and Tanaka, 1987), through the treatment of RBCs for 24 hours with two different concentrations (4mM and 8mM in cRPMI) of sodium fluoride (NaF), similar to the described by Ayi et al. (2009). RBCs were incubated with NaF for 24 hours, then washed and put in newly prepared cRPMI and ATP level was daily measured, for 96 hours. The maintenance of PKD phenotype for 96 hours after NaF withdrawal was confirmed in a previous experiment, by daily ATP level measurements, using the ATP cell viability luciferase assay kit (Sigma-Aldrich), according to manufacturer’s orientation (data not shown). The amount of light emitted was measured as previously described and ATP (pmol) was quantified through a linear regression calibration equation performed with known ATP concentrations. Since the amount of ATP depleted with 2mM and 4mM NaF was slightly lower than that described by Ayi et al. (2009), we chose to work with 4mM and 8mM NaF. Incubation occurred before infection to assure no effect on parasite enolase (Pal Bhowmick et al., 2009).

In summary, five experimental groups were simultaneously analyzed in the invasion/maturation assay: 1) RBCs incubated for 24 hours with 2,3-DPG 8mM prior to infection and then, put in culture conditions (iRBC DPGt), 2) infected RBCs incubated with 2,3-DPG 8mM added every 24 hours to culture medium after infection (iRBC DPGadd), 3) and 4) two different infected NaF-inducible PKD RBCs described above (iRBC NaF4 and iRBC NaF8 respectively), and 5) a control group with infected RBCs in cRPMI alone (iRBC C).

ATP depletion inside RBCs due to 2,3-DPG and NaF treatment was assessed by ATP level measurements in the five experimental groups and respective non-infected controls added for each condition (niRBC DPGt, niRBCadd, niRBC NaF4, niRBC NaF8 and niRBC C). Measures were done at the time-points conventionally named -24h (at the end of non-infected RBC incubation with NaF, 2,3-DPG or cRPMI alone for 24h), -10h (removal of compounds by washing with cRPMI), at 0h (onset of invasion and maturation assays) and every 24 hours afterwards for 96 hours. Measurements were done using the same kit as above and performed in triplicate, both in infected-RBCs and in paired control non-infected RBCs, kept exactly under the same conditions as described above.

#### 2.4.4 Egress Assay

An egress assay was designed to evaluate the effect of 2,3-DPG on the parasite ability to egress from an infected and 2,3-DPG treated RBC and start a new cycle by reinvading untreated RBCs.

Initial ring-stage synchronized *P. falciparum* cultures were allowed to grow for a full cycle, being then divided into four new cultures with a 5% hematocrit and 1% ring stage parasites. 2,3-DPG 8mM was added to the culture medium of two of these experimental groups and all four were incubated at standard conditions with daily media change. At approximately 44 hours of the parasite life cycle, schizonts from one culture treated with 2,3-DPG and from an untreated one were recovered through magnetic separation using LD columns attached to a QuadroMACS separator (Miltenyi Biotec), while other two cultures were allowed to grow under normal conditions. After magnetic separation, at approximately 48 hours (mature schizonts stage), three similar 96 well flat-bottom microplates were then prepared as follows: 1) 100μL triplicates of recovered schizonts from the two enriched cultures were added to untreated RBCs (niRBCs C) to a final 3% hematocrit and 1% schizont density and 2) 100μL triplicates from non-enriched cultures were cultivated at the same 3% hematocrit.

Plates were incubated for parasite reinvasion at standard conditions and parasite densities were read by flow cytometry (Cytoflex, Beckman Coulter) at approximately 48 hours of the current parasite life cycle and then at 1.5, 3, and 24 hours of the following cycle, i.e., after invasion. For each plate reading, after incubation, SYBR Green solution (0.001% v/v in PBS) as described above was added to each cell. The plate was incubated again for 45 minutes in standard conditions, centrifuged the supernatant was discarded, and the cells were resuspended in 100μl of 1χ PBS. Hematocrits were diluted to 0.3% in new wells. Three independent assays were performed. Approximately 100,000 RBCs were analyzed, and parasite density was calculated using FlowJo V10 software (Tree Start Inc.).

### 2.5 Untargeted Metabolomics Analysis

Synchronized *P. falciparum* cultures at ring-stage forms of 12 hours post-invasion and cultures of non-infected RBCs submitted to the same synchronization procedure using 5% sorbitol, as described above were cultivated in cRPMI supplemented or not with 2,3-DPG 8mM for 30 hours. Four biological replicates to each experimental condition corresponding to non-infected and infected-*P. falciparum* RBCs, treated or untreated with 2,3-DPG were used.

Although the RBC is a simple anucleate cell with well-characterized metabolic pathways and 2,3-BPG targets and effects inside RBC are known, an untargeted metabolomics analysis was performed as a first approach to provide an unbiased analysis and the possibility of expose new targets or biomarkers of 2,3-DPG treatment on parasites.

#### 2.5.1 Extraction of Metabolites

Metabolites were extracted from each biological replicate according to a protocol developed by MacRae et al. (2013) with little adjustments. Briefly, after 30 hours of *in vitro* culture non-infected and infected-RBCs, treated with 2,3-DPG or untreated, were quickly quenched from 37°C to 12°C through immersing Falcon tubes containing the cultures for few seconds, in a solution of metallic beads in absolute ethanol kept overnight at -80°C. After that, all the downstream procedures were performed at 4°C. Infected treated or untreated RBCs were subsequently submitted to a magnetic separation procedure to deplete non-infected RBCs and assure metabolite extraction from an enriched material comprised with more than 95% of infected RBCs by mature forms. Thus, 3.05 χ 10^7^ RBCs per biological replicate were submitted to extraction in 1.5 mL Eppendorf microtubes. A solution with chloroform and methanol (2:1 v/v) was added into each microtube and then immersed in water/ice solution in an ultrasound cleaning bath (Model 3000512, Voltage 220V, Current 0.2A, Power 50W, JP Selecta) and submitted to three series of eight minutes of high frequency sound waves (40kHz) interpolated by 12 minutes of resting, for one hour. The liquid content was removed from each microtube and concentrated in new ones using a concentrator device (5301 speed-vac Eppendorf) at 250χ*g* and room temperature. Then, pellets were resuspended in a solution with methanol and water (2:1 v/v) and submitted to a new round of high frequency sound waves as described above. The liquid content was removed and added to the dried material inside the previous microtubes, and a new round of concentration was performed. Finally, a solution with chloroform, methanol, and water (1:3:3 v/v/v) was added to the dried material in each microtube, then vortexed rigorously and centrifuged for 5 minutes at 0°C at 20817χ*g*. The upper phase containing polar metabolites was separated from the lower phase containing apolar metabolites, both phases were concentrated in separated tubes in the speed-vac and stored at -80°C until high performance liquid chromatography coupled to mass spectrometry (HPLC/MS) analysis.

#### 2.5.2 High Performance Liquid Chromatography Coupled to Mass Spectrometry (HPLC/MS)

Samples were analyzed by liquid chromatography (Ultimate 3000 RSLC nano system, Thermo Fisher Scientific) interfaced with a Bruker Impact II quadrupole time-of-flight mass spectrometer equipped with an electrospray source (Bruker Daltoniks, Bremen). Chromatographic separation was performed on a column Kinetex Polar C18, 2.6µm, 100Å, and 100 × 2.1mm (Phenomenex) at 45°C. The gradient was as follows: 1 min 5% phase B, then in 5 min to 50% phase B, next in 4 min to 100% phase B, held for 8 min at 100% phase B, subsequently in 1 min to 0% phase B and held for 6 min at 0% phase B. Internal calibration was achieved with a sodium formate solution introduced to the ion source *via* a 20μL loop at the beginning of each analysis using a six-port valve. Calibration was then performed using high-precision calibration mode (HPC). The Mass Spectrometer was operated in positive ionization mode on the full scan mode and data was acquired in the mass range from m/z 50 to 1000 with a spectra rate of 1 Hz. The capillary was set at 4500 V, the End Plate offset at 500 V, the Nebulizer gas (N2) at 4 Bar and the Dry gas (N2) at 8 L/min at 200°C. To evaluate the performance of the instrument, quality control pools (QCpool, n=3; made by pooling 5µl of upper and lower extracted phases) were injected every 7 to 10 samples.

#### 2.5.3 Data Extraction

LC-MS files (31 samples, one infected treated sample from upper phase was lost during the LC-MS process, 3 QCpools and 4 water samples) were converted to mzXML files using the ProteoWizard MSConvert software (Chambers et al., 2012). LC-MS data was then preprocessed using XCMS (R package xcms 3.6.2) (Smith et al., 2006; Tautenhahn et al., 2008; Benton et al., 2010) and consisted of peak picking, retention time alignment, peak matching and gap filling. Peak picking was performed with the centwave algorithm and the following parameters: ppm=15, peakwidth = 10-40 s, snthresh = 10, prefilter = 3, 10,000 and noise=1000. Retention time alignment was performed against the average of the QCpools with a m/z width = 0.01. Peak grouping was performed with the following parameters: bandwidth = 5, m/z width = 0.01, minimum fraction = 0.5. Gap filling was performed with a fixed retention time deviation = 10 s. A total of 356 features (peaks with specific retention time and *m/z* values) were extracted with their corresponding m/z, retention time and peak area.

In order to reduce the number of features, two filters were applied. The first filter excluded all ions that appeared in water samples with an average area > 10,000. The second filter excluded all ions with a coefficient of variability >= 30% in the QC pools. After applying these filters, the list was reduced to 90 features. Data was normalized by total area.

#### 2.5.4 Metabolite Identification

After statistical analysis, MS and MS/MS data of ions of interest were manually searched against metabolomics databases (HMDB and Metlin). SIRIUS metabolomics search tool was also used (Dührkop et al., 2019).

### 2.6 Statistical Analysis

Statistical analysis was performed using GraphPad Prism version 8 for Windows (GraphPad Software). Kolmogorov-Smirnov test was used to assess data normality and non-parametric Mann-Whitney U test, or unpaired Student’s t test was used for comparisons on continuous values between two-independent groups, as appropriate.

Intergroup comparisons were conducted for the merozoite count assay, through the application of unpaired Student’s t test between treated and untreated sample groups.

For metabolomics statistical analyses, LC-MS data was centered, and unit-variance scaled. Metabolic differences between non-infected and infected-*P. falciparum* RBCs, both treated and untreated, were assessed using multivariate analysis based on principal component analyses (PCA) and partial-least squares discriminant analyses (PLS-DA) using SIMCA software (MKS Umetrics, Sweden Umetrics, version 16.0.1). Venn diagrams were built in R environment (https://cran.r-project.org/bin/windows/base/old/3.6.1) using the “VennDiagram” package.

For all tests, statistical significance level was set at <0.05, unless otherwise stated.

### 2.7 Ethics Statement

This study followed the European and national directives. All blood donors were clearly informed that participation in the study was voluntary and confidential and were made aware of the objectives of the work. Each participant signed an informed consent form before blood collection and a numerical code was assigned to each donor to maintain confidentiality. The Ethical Committee of IHMT approved the protocol (as part of project PTDC_BIA-CEL_28456_2017, *Parecer* 16.18).

## 3 Results

### 3.1 2,3-DPG Has Little Effect on Human Cells

No hemolysis was observed due to the direct effect of 2,3-DPG for any of the doses added to the culture medium comprised in the range of two-fold serial dilutions from 16mM to 0.25mM or to an indirect effect of sodium concentration or changes in pH value of the solutions for 90 minutes of incubation as opposed to the positive control. This observation was confirmed during all the subsequent experiments where no hemolysis occurred with this same range of serial dilutions of 2,3-DPG concentrations administrated up to 96 hours with daily media renewal to in *in vitro* cultures of infected or non-infected red blood cells (data not shown).

On cytotoxicity assay, HEPG2 viability started to decrease from the 2,3-DPG dose of 4mM and less than 10% of viable cells were observed with the 16mM dose. The IC50, the minimum concentration of 2,3-DPG able to inhibit the cellular growth or reduce the cell viability in 50%, was 6.40mM (95% CI of IC50 5.16-7.78). Results of three independent experiments are summarized in **Supplementary Figure S1**.

### 3.2 Effect of 2,3-DPG Addition on Parasite Development

#### 3.2.1 2,3-DPG Showed Antimalarial Activity Causing a Significant Decrease of Parasite Densities

IC50 values determined at time points of 24h, 36h, 48h, 60h, 72h and 96h are presented in **Table 1** and **Supplementary Figure S2** (it was not possible to determine the values at 12h and 84h). As a concentration of 8mM would be well tolerated by the RBCs (physiological concentration at high altitudes) and had some effect on the parasite (IC50), we considered this dose for all the following assays.

**Table 1:** IC50 values after 24, 36, 48, 60, 72 and 96 hours of incubation of 3D7 *P. falciparum* parasites with 2,3-DPG.

Time point (hour)	24	36	48	60	72	96
IC50 (mM) ± SD	3.42 ± 1.48	6.21 ± 0.80	8.56 ± 0.33	8.18 ± 0.80	5.61 ± 0.59	10.17 ± 1.95

**Figure 1:**
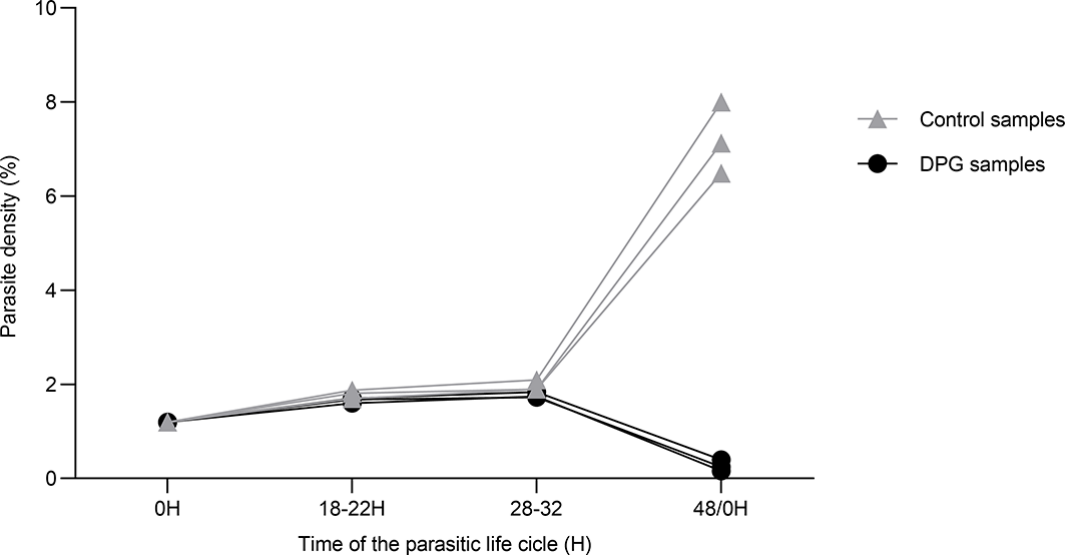
2,3-DPG caused a significant decrease of parasite densities. Total parasite density of 3D7 *P. falciparum* parasites during one cycle of growth (48h) in the absence (**light triangles**) and presence (**dark dots**) of 2,3-DPG 8mM. This graph was chosen as representative of three independent experiments performed in triplicate.

We observed that the addition of 2,3-DPG 8mM into culture medium impairs the parasite growth as shown in **Figure 1**. For the three independent experiments, which have started with initial parasite density of 1%, results show that, under normal conditions, there is an exponential increase in density levels from approximately 30h to 48h/0h, corresponding to the parasites egress from the host cell and invasion of new RBCs to start a new asexual cycle. On the opposite, when parasites have grown in the presence of 2,3-DPG for 48 hours, parasite density levels decreased at the same time point, suggesting that parasites are able to mature but cannot normally progress into a new cycle.

#### 3.2.2 2,3-DPG Impacts Parasite Development But Does Not Deplete ATP

**Figure 2:**
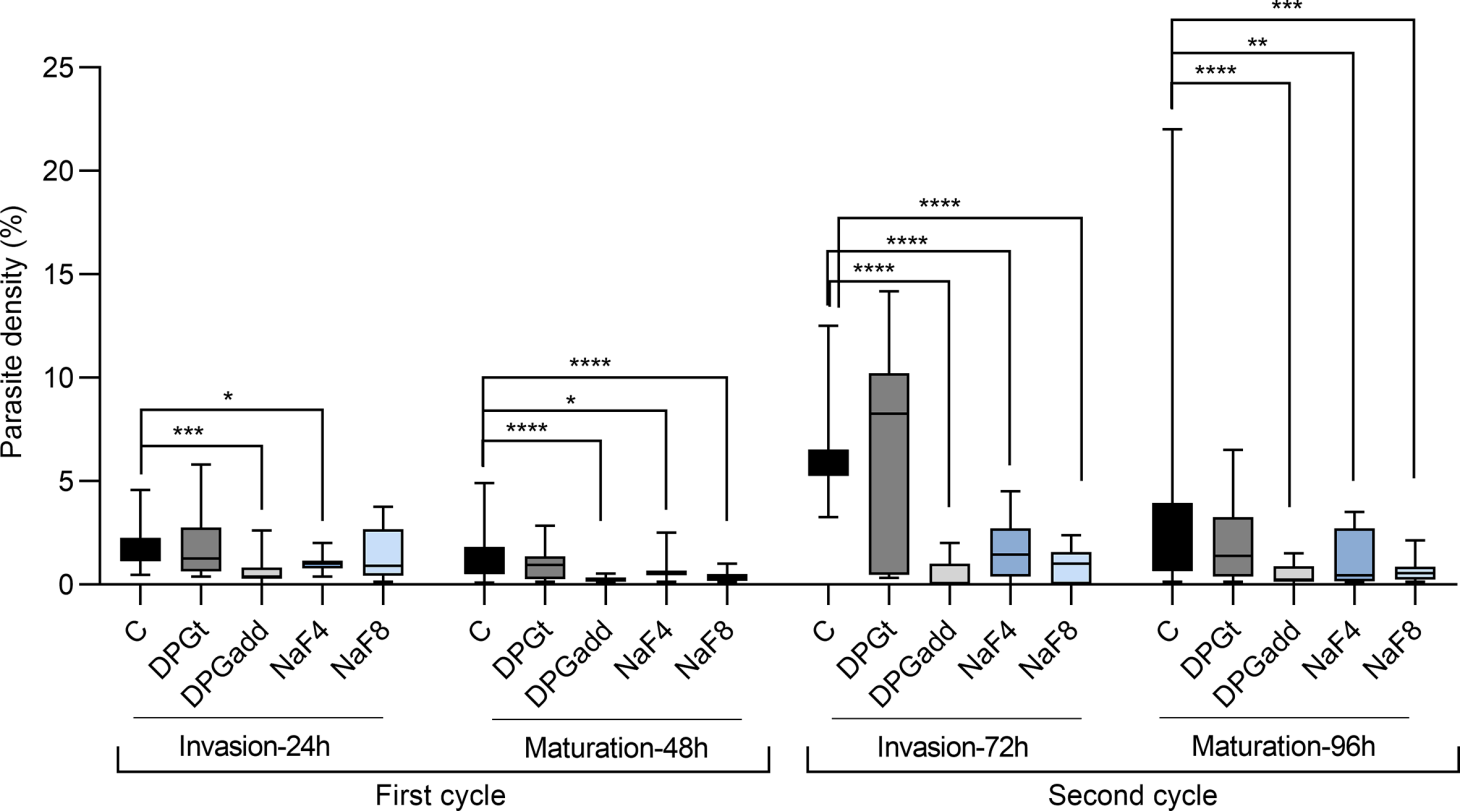
Impact on parasite development. Parasite densities measured in 3D7 *P. falciparum* parasites during invasion and maturation assays for two growth cycles (96h). Invasion of erythrocytes was assessed at 24 hours (1st cycle) and 72 hours (2nd cycle), and maturation was assessed at 48 hours (1st cycle) and 96 hours (2nd cycle). Data are summarized from three independent experiments. C - control group with parasites growing in cRPMI alone, DPGt - RBCs incubated for 24 hours with 2,3-DPG 8mM prior to infection, DPGadd - 2,3-DPG 8mM added every 24 hours to culture medium, NaF4 and NaF8 - inducible-PKD RBC groups, incubated with NaF 4mM and 8mM, respectively (*p < 0.05, **p < 0.01, ***p < 0.001, ****p < 0.0001; lines inside boxes represent median values and error bars the minimum and maximum values for each box).

**Figure 2** summarizes data of parasite densities from three independent experiments where the effect of the addition of 2,3-DPG (prior infection or daily added) and of PKD phenotype induction, on parasite invasion (at 24h – first cycle, and at 72h – second cycle) and maturation (at 48h – first cycle, and at 96h – second cycle) was assessed.

Parasite densities over two parasite cycles were significantly lower than the control group when RBCs were previously treated with NaF 4mM and 8mM (NaF4 and NaF8, respectively) or when culture medium was daily supplemented with 2,3-DPG 8mM (DPGadd). Parasite densities started to differ at 24 hours and become more notorious at 48 and 72 hours (predominance of schizont and ring stages, respectively). On the opposite, when RBCs were previously treated with 2,3-DPG 8mM for 24 hours before being infected (DPGt), similar parasite densities to the control group were observed.

**Figure 3:**
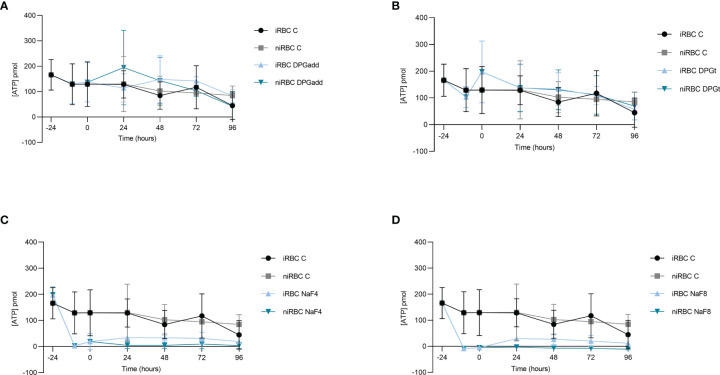
2,3-DPG does not deplete ATP. ATP content inside RBCs under different experimental conditions tested during the invasion and maturation assay. **(A)** 2,3-DPG 8mM added every 24 hours to culture medium, infected (iRBC DPGadd) and non-infected (niRBC DPGadd), **(B)** RBCs incubated for 24 hours with 2,3-DPG 8mM prior to infection, infected (iRBC DPGt) and non-infected (niRBC DPGt), **(C)** inducible-PKD RBC incubated with NaF 4mM, infected (iRBC NaF4) and non-infected (niRBC NaF4); **(D)** inducible-PKD RBC incubated with NaF 8mM, infected (iRBC NaF8) and non-infected (niRBC NaF8). Untreated RBCs, infected (iRBC C) and non-infected (niRBC C) control groups were included in all experiments. Error bars represent the standard deviation in each timepoint.

ATP concentration inside RBCs was measured in the different experimental groups both in infected-RBCs and paired non-infected controls at the time of incubation and removal of 2,3-DPG and NaF (-24 hours and -10 hours, respectively), at the onset of invasion and maturation assay (0 hours) and every 24 hours onwards for 96 hours (**Figure 3**). A depletion of ATP was only observed when RBCs were previously incubated with NaF being the concentration slightly higher in the infected cells. 2,3-DPG addition, either previously to infection or daily to the culture medium, caused no effect on the ATP levels in the cells, independently of infection.

#### 3.2.3 2,3-DPG Does Not Hinder Parasite Egress But Reduces Progeny Number

Although no hemolysis had been observed or ATP depletion as a result of 2,3-DPG addition, we cannot exclude an adverse effect on the RBC membrane, which could prevent parasite progression into a new cycle. Therefore, the ability of parasites that have grown in the presence of 2,3-DPG 8mM added daily to culture medium to egress from RBCs and start a new intraerythrocytic cycle by reinvading untreated cells (niRBCs C) was assessed through an egress assay. Both enriched (schizont stage) and non-enriched parasites, from treated and untreated cultures, were allowed to pursue the developmental cycle in untreated RBCs.

As previously shown, parasite densities were higher in untreated cultures than in cultures treated with 2,3-DPG 8mM for 48 hours, which was more evident in non-enriched cultures (Untreated Normal *versus* DPG Normal, **Supplementary Figure S3**). Nonetheless, a similar parasite growth rate in the new cycle in untreated cells was observed in all four groups, regardless of the previous treatment with 2,3-DPG.

**Figure 4:**
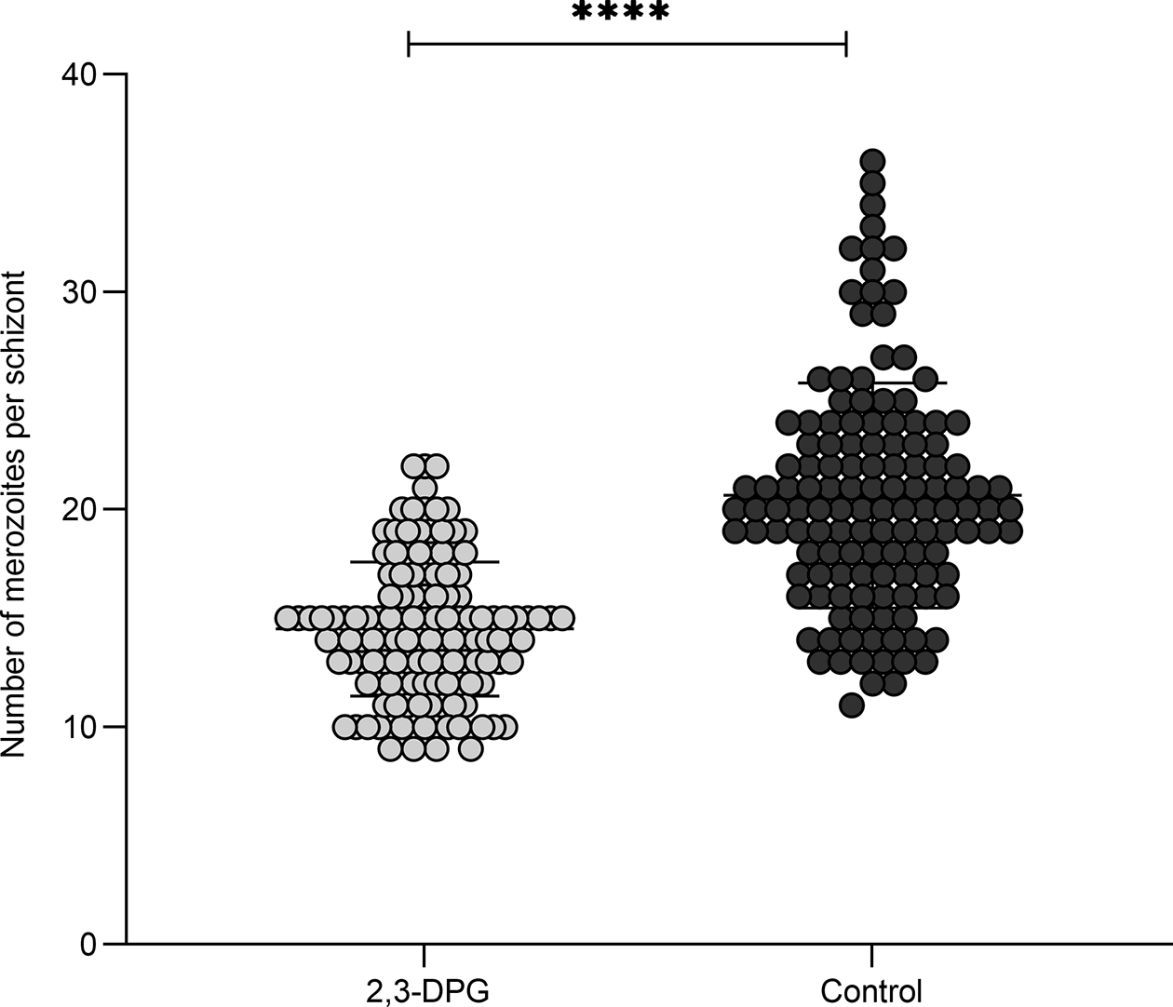
2,3-DPG reduces progeny number. Merozoite count within late-stage schizonts growing in presence or absence of 2,3-DPG 8mM. n=132 schizonts; Presence (light dots, median = 14.00, mean 14.51, SD 3.075) or absence (dark dots, median = 20.00, mean 20.65, SD 5.169) of 2,3-DPG. ****p < 0.0001.

As the egress or invasion of new cells did not seem compromised, we analyzed by light microscopy, whether the number of resulting merozoites from parasite segmentation process (schizogony) could have been altered due to effect of 2,3-DPG treatment, impacting the parasite multiplication and increase of parasite densities, by counting the number of merozoites present within intact, late-stage schizonts from both 2,3-DPG treated and untreated RBCs (n=132 each). **Figure 4** shows a scatter plot where each point represents the number of merozoites within a single schizont that either developed in the presence or absence of 2,3-DPG, showing a significant difference between samples (p value<0.0001). Schizonts that developed in the presence of the compound ultimately resulted in a lower number of merozoites (mean=14.51; SD=3.08) than those that have grown in normal culture medium (mean=20.65, SD=5.17). The same trend was obtained with the inducible-PKD groups, but the low parasite densities precluded a similar analysis.

### 3.3 2,3-DPG Makes the Metabolic Profile of an Infected Cell More Similar to That of a Non-Infected Cell

PCA was performed with all samples and QCpools to identify which of the factors (sample phase, infection, or treatment) had the most important influence on the metabolic profile. The first two components explained 47% and 24% of the variance of the data, respectively. Sample phase was the most relevant factor, as there was a clear separation between lower and upper phases (**Supplementary Figure S4**). In addition, the variability of the samples was much higher than the variability of the QCpools, confirming the LC-MS analysis performance.

**Figure 5:**
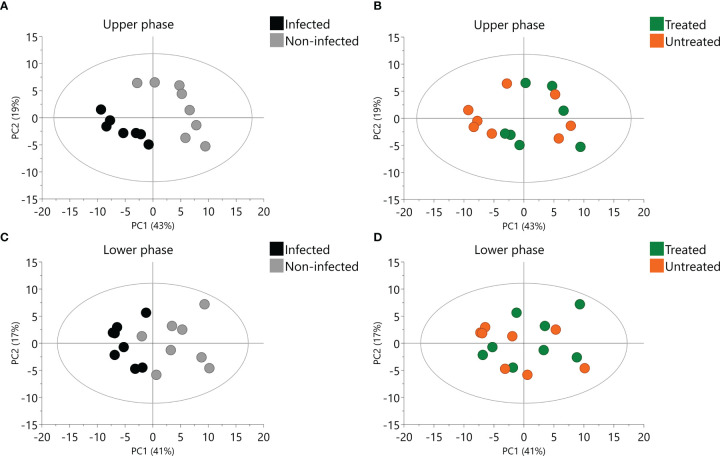
Principal Component Analysis (PCA) of upper and lower phase samples. Scores plot of upper phase samples colored according to infection **(A)** or 2,3-DPG **(B)**. Scores plot of lower phase samples colored according to infection **(C)** or 2,3-DPG **(D)**.

PCA were performed for all upper and lower phase samples separately. The first and second components of the models explained 41% and 17% of the variance of the lower phase data and 43% and 19% of the variance of the upper phase data. Infection had a clear influence in the metabolic profile, independently of the phase (**Figures 5A, C**). Influence of 2,3-DPG exposure was less clear (**Figures 5B, D**). However, treated infected erythrocytes appeared closer to non-infected erythrocytes, mainly in the upper phase samples (**Figure 5B**).

**Figure 6:**
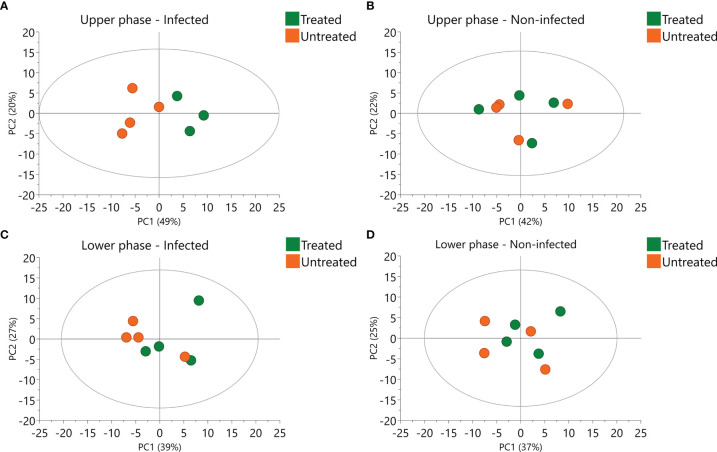
Principal Component Analysis (PCA) of infected and non-infected samples. Scores plot of infected **(A)** and non-infected **(B)** upper phase samples colored according to 2,3-DPG. Scores plot of infected **(C)** and non-infected **(D)** lower phase samples colored according to 2,3-DPG.

In order to better evaluate the influence of 2,3-DPG, PCAwere performed in infected (**Figures 6A, C**) and non-infected samples (**Figures 6B, D**), separately, for each phase. Influence of 2,3-DPG was only observed in infected samples for both upper and lower phases (**Figures 6A, C**, respectively).

Since the aim of the untargeted metabolomics analyses was to evaluate the metabolites that were affected by 2,3-DPG in infected RBCs, the first approach was to build PLS-DA models between treated and untreated RBCs. However, no statistically significant models were obtained either considering all samples or only infected or non-infected samples for both upper and lower phases. One of the possible causes of no statistical significance might be the reduced number of infected treated samples (n=3 for the upper phase). Taking this in consideration together with the proximity of infected treated RBCs to non-infected RBCs observed in the PCA (**Figure 5B**), we built a PLS-DA model to compare infected treated samples and non-infected samples (treated and untreated) versus infected untreated samples (p = 0.005, R2(X) = 0.43, R2(Y) = 0.66, Q2(Y) = 0.58; upper phase samples). We also built a PLS-DA model comparing only non-infected samples (treated and untreated) versus infected untreated samples (p = 0.002, R2(X) = 0.71, R2(Y) = 0.99, Q2(Y) = 0.96; upper phase samples). By comparing the most relevant features (peaks with a specific retention time and m/z value) of both models, we can disclose those features that are specific for the effects of 2,3-DPG on infected cells. The most relevant metabolites for each model were defined as those having a Variable Importance on the Projection (VIP) value > 1 and a correlation coefficient p(corr) value > |0.6|. A total of 31 features were obtained in each model, from which 27 were common and 4 were specific for each model (see Venn-Diagram in **Supplementary Figure S5**). The four features that were specific for the PLS-DA model comparing infected treated and non-infected (treated and untreated) samples *versus* infected untreated samples can be considered as being specific for the effects of 2,3-DPG on infected cells, since they are not relevant in the PLS-DA model that compares non-infected samples (treated and untreated) *versus* infected untreated samples. Whereas we could not identify any of the four features, their *m/z* ratios are presented in the **Supplementary Figure S5**.

## 4 Discussion

With a complex life cycle, the malaria parasite has interacted with the human host for thousands of years, and this coevolution has shaped both parasites and humans. Malaria has been one of the strongest selective forces on the human genome and has selected many human genetic variants that would hardly be maintained in human populations due to their impact on human health. Nevertheless, these variants, most of them related to erythrocyte specific structural proteins or enzymes, may reach high frequencies in malaria endemic areas as they provide some protection against malaria disease (Kwiatkowski, 2005).

Among erythrocyte enzymopathies, the association between G6PD deficiency and protection against malaria is the best described (Mbanefo et al., 2017). The second most frequent erythrocyte enzymopathy worldwide is PK deficiency, but only a few studies addressed the frequency of PK-deficiency in malaria endemic areas and its possible association to malaria protection (Alves et al., 2010; Machado et al., 2010; Berghout et al., 2012; Machado et al., 2012; Bruggen et al., 2015). Still, as RBCs do not have nucleus and cannot produce new proteins to replace old or depleted ones, any limitation in the erythrocyte metabolic pathways may carry consequences to the cell integrity and may interfere with parasite development. Some evidence gathered from experimental models shows that this is the case of PK deficiency. The present study aimed to further explore the mechanisms involved in this interaction, focusing on the role of 2,3-BPG in PK deficiency-mediated protection against malaria parasites.

The malaria parasite *P. falciparum* relies on glycolysis for the generation of energy and holds a complete set of glycolytic enzymes, except BPGM, that seems to differ biochemically and structurally from their host counterparts (Roth, 1990). The rate of glycolysis in *P. falciparum*-infected erythrocytes is significant, using glucose and generating lactate at 20 to 100 times the rate observed in non-infected cells (Mehta et al., 2005; Schalkwyk et al., 2008). Pyruvate kinase activity is a rate-limiting step of RBC glycolysis (Roy et al., 2021), and a relative increase in its activity rises RBC ATP at the expense of 2,3-BPG. Under normal circumstances, the parasite seems to be able to operate its own PK isozyme into the host red cells and an increase in RBC ATP and a decrease in 2,3-BPG are observed in infected cells, which results from a PK activity with parasite origin (Oelshlegel et al., 1975; Dubey et al., 2003), also suggesting that the enormous glucose flux into the infected RBCs is almost totally for parasite use. However, when host PK activity sharply decreases, 2,3-BPG content rises, and we questioned whether it could have harmful consequences for the parasite. We analyzed an induced PK-deficiency phenotype (through enolase inhibition and confirmed by the depletion of ATP) and the effect on the parasite if 2,3-BPG itself (synthetic form 2,3-DPG) would be administered in the extracellular medium.

Present results show that at the end of an intraerythrocytic developmental cycle, when 2,3-DPG 8mM was daily added into culture medium, there was a sharp reduction in parasite densities and parasites were not able to normally progress to a new cycle. We analyzed this impairment of parasite growth through invasion and maturation assays, and we observed that parasite densities were significantly lower than the control group and this effect is equally visible both on invasion and maturation. Same result is obtained with the PK-deficiency induced phenotype, which suggests a similar effect and a role for the 2,3-DPG. However, this mechanism seems not to be related with the intracellular level of ATP as an ATP depletion, which is also a consequence of PK-deficiency and may also be damaging for the parasite (Min-Oo et al., 2003; Ayi et al., 2009), was only observed in the PK-deficiency induced phenotype, being the concentration slightly higher in the infected cells which may be due to the ATP production by the parasite (Jacobasch et al., 1990).

The cycles of growth that result in parasite proliferation depend both on the ability of the parasite to access and complete invasion of susceptible cells as well as to mature and generate new merozoites to egress from the host cell. The egress from RBCs and the normal progression for a new intraerythrocytic developmental cycle was confirmed, being the sharp reduction on parasite densities due to a direct effect on parasite maturation as significant lower progeny emerged from parasites that were submitted to 2,3-DPG. Parasite may adapt its multiplication and regulation of progeny number as a result of nutrient exhaustion or type or stress factors (Mancio-Silva et al., 2017; Simon et al., 2021), and also may be influenced by intrinsic or extrinsic host factors as mimicked in the present study.

Several glycolytic enzymes from human erythrocytes such as hexokinase, phosphofructokinase, pyruvate kinase, and phosphoglycerate kinase are inhibited by unbound 2,3-biphosphoglycerate, as a regulatory effect on glycolytic pathway (Ponce et al., 1971). As previously stated, *Plasmodium* parasite has a complete set of glycolytic enzymes. The addition of 2-halo derivatives of D-glucose results in the rapid acidification of the parasite cytosol, inhibition of glucose phosphorylation by hexokinase, and a marked reduction in the ATP levels within the parasite having an effect on the parasite proliferation (van Schalkwyk et al., 2008). The synthetic compound 2,3-DPG may be acting as an inhibitor of parasite key enzymes of glycolysis such as hexokinase, phosphofructokinase, pyruvate kinase, and phosphoglycerate kinase (Roth et al., 1988). As we did not observe any impact on ATP production in infected RBCs, maybe there would be another target of 2,3-DPG on the parasite. The results obtained with our PLS-DA model comparing infected (treated) and non-infected (treated and untreated) samples versus infected untreated samples for polar metabolites (upper phase samples) suggest new targets and we cannot exclude novel and undescribed parasite metabolites as 2,3-DPG targets. The analysis of the parasite’s metabolic capabilities is likely to unveil these presumable effects.

The addition of 2,3-DPG seemed to not affect the host cell as no change on ATP levels were observed, independently of infection, and no effect on parasite was obtained when only RBCs were treated prior to infection. The effect only on the parasite was also suggested by the closer metabolic profile of infected and treated RBC to the one from non-infected cells, as observed with the untargeted metabolomics analysis. 2,3-DPG treatment appeared not only not to affect the RBC, but to reverse the metabolic profile of the infected cell and make it closer to the non-infected. Unfortunately, metabolomics analysis was not conclusive to determine which metabolites could be differentially present in infected treated RBCs that could restore the metabolic profile to the one of non-infected RBCs. A future approach with biological replicates with higher parasite density and complemented with targeted analyses addressing the four polar metabolites seen in the present study will contribute to elucidate the impact of 2,3-DPG on metabolic profile of infected RBCs.

The targeted action of 2,3-DPG on parasite rather than on host cell may be due to the differences between the membrane of the infected and non-infected erythrocytes as the parasite metabolic activity results in an increased permeability of the infected erythrocyte membrane (Mehta et al., 2006; Desai, 2014; Counihan et al., 2021), which may facilitate the 2,3-DPG entrance into the cell. However, since increased amounts of intracellular 2,3-BPG affect the mechanical stability of RBC membrane, an effect on host cell cannot be excluded and needs further analyses.

Targeting host glycolysis as an antimalarial strategy is a promising approach and has been attempted in a few studies. Jezewski et al. (2021) observed that enolase inhibition increased erythrocyte susceptibility to oxidative damage and induces rapid and premature erythrocyte senescence, rather than direct hemolysis, also affecting parasite development. However, an inhibitory effect also on parasite enolase was not unequivocally ruled out, so the interaction mechanism was not clearly established. Xu et al. (2020) reported that mice suffering from BPGM deficiency, the enzyme responsible for the synthesis of 2,3-BPG, show severely reduced 2,3-BPG levels and protection against severe malaria-induced anemia and intensity of neuroinflammation on experimental cerebral malaria. The authors suggest that the reduced 2,3-BPG levels results in increased O_2_-bound hemoglobin, decreased oxygen delivery to tissues and compensatory erythropoiesis, which leads to protection against anemia. This augmented erythropoiesis response to mounting parasitemia and RBC destruction would provide more efficient replacement of infected RBCs. Further, the authors observed a 5% decrease in the proportion of *Plasmodium* late-stage parasites in deficient RBCs and suggest that intracellular environment may be less permissive to replication and maturation of the malarial parasite. This would be contrary to our observations, but authors cannot exclude that RBCs infected with mature parasite forms are being more efficiently replaced in the process of superior erythropoiesis.

Oppositely, Roth et al. (1988) have grown *P. falciparum* parasites in RBCs lacking BPGM, where 2,3-BPG metabolite was barely detectable and parasite growth was entirely normal. Mehta et al. (2005) demonstrated a downregulation of the glucose utilization rate in the majority of non-infected RBCs by the infected ones, suggesting a selective inhibition of non-infected RBCs’ phosphofructokinase due to a decrease in pH as a result of production of lactic acid by the infected cells. Authors suggest that reduced glucose utilization would result in lower ATP production in erythrocytes and lower 2,3-BPG levels with subsequent lower oxygen supply to the tissues, contributing to an overall lethargic state for the patient.

A better understanding of the above mechanisms may provide potential targets for new approaches such as development of host directed therapies (HDT), which could potentially be designed to mimic the protective effects exhibited by naturally occurring RBC disorders (Zumla et al., 2016), allowing a better management of complicated and uncomplicated malaria patients. Despite this not being a therapeutic study, the observations that glycolysis, namely the possible action of an increased amount of the 2,3-BPG metabolite, may be important for this goal. Its action on the reduction of the intraerythrocytic multiplication of *P. falciparum* may help to delay the development of life-threatening parasite densities until parasite clearing immunity or the effect of a co-delivered traditional antimalarial are achieved, transcending multidrug resistance. However, the mechanism underlying this effect remains unclear and further work will be carried out in order to fully disclosure the impact on the parasite and overcome the major limitations of the present study, namely an unequivocal quantification of the amount of 2,3-DPG inside the cell and the inconclusive results of the pilot metabolomics analysis of infected and non-infected cells submitted to the action of the compound since it was not possible to identify the extracted metabolites nor to detect the compound inside the cell.

## Data Availability Statement

The original contributions presented in the study are included in the article/**Supplementary Material**. Further inquiries can be directed to the corresponding author.

## Ethics Statement

The studies involving human participants were reviewed and approved by Ethical Committee of Institute of Hygiene and Tropical Medicine, University NOVA of Lisboa, Lisbon, Portugal. The patients/participants provided their written informed consent to participate in this study.

## Author Contributions

All authors have read and agreed to the published version of the manuscript. Conceptualization: APA. Methodology: JM, SMT, FN, JAR, and FAC. Formal analysis: IM, MMM, MC, JM, SMT, AMMA, and APA. Funding acquisition: APA and FAC. Investigation: IM, MMM, MC, SMT, JN, AB, and MTGR. Project administration: APA. Supervision: APA. Writing—original draft: IM, MMM, MC, JM, and APA. Writing—review and editing: MTGR and AMMA. All authors contributed to the article and approved the submitted version.

## Funding

This research was funded by Fundação para a Ciência e Tecnologia (https://www.fct.pt/index.phtml.en), projects PTDC_BIA-CEL_28456_2017 and GHTM - UID/04413/2020 (https://ghtm.ihmt.unl.pt/). The funders had no role in study design, data collection and analysis, decision to publish, or preparation of the manuscript.

## Conflict of Interest

Author JAR was employed by company Clarify Analytical.

The remaining authors declare that the research was conducted in the absence of any commercial or financial relationships that could be construed as a potential conflict of interest.

## Publisher’s Note

All claims expressed in this article are solely those of the authors and do not necessarily represent those of their affiliated organizations, or those of the publisher, the editors and the reviewers. Any product that may be evaluated in this article, or claim that may be made by its manufacturer, is not guaranteed or endorsed by the publisher.
