# Supplementary material for: Synthetic Red Blood Cell-Specific Glycolytic Intermediate 2,3-Diphosphoglycerate (2,3-DPG) Inhibits Plasmodium falciparum Development In Vitro
Source: Front Cell Infect Microbiol. 2022 Mar 15;12:840968. doi: 10.3389/fcimb.2022.840968 (PMC8967366; doi:10.3389/fcimb.2022.840968)
Supplement: Supplementary file 1 [file DataSheet_1.zip › suplementary material/Supplementary Material Captions.docx]

**Synthetic red blood cell-specific glycolytic intermediate 2,3-diphosphoglycerate (2,3-DPG) inhibits *Plasmodium falciparum* development *in vitro***

# Supplementary material Captions

**Figure S1**. **Effect of 2,3-DPG on human cells: toxicity assay**. A log dose-response curve was obtained for HepG2 cells incubated with two-fold serial dilutions of 2,3-DPG concentrations (0.25 to 16mM) for 48 hours, following a subsequent incubation with MTT compound. Cell viability was compromised after 48 hours under the effect of 2,3-DPG 4mM (orange line, corresponding to 80% of viable cells) and was almost totally compromised after 48 hours under 2,3-DPG 16mM (blue line). IC50 value is shown in the graph corresponding to a 2,3-DPG dose of 6.04mM and the thick lines correspond to extreme values of IC50 95% Confidence Interval, i.e., 2,3-DPG doses of 5.16mM and 7.78mM. Error bars represent standard deviation values for % of viable cells for each 2,3-DPG concentration.

**Figure S2.** **Log dose-response curves for 3D7 *P. falciparum* parasites** incubated with two-fold serial dilutions of 2,3-DPG concentrations (from 0.25 to 16mM) for **A)** 24 hours, **B)** 36 hours, **C)** 48 hours, **D)** 60 hours, **E)** 72 hours, **F)** 96 hours. Different lines correspond to three different assays. Error bars represent the standard deviation in each sample.

**Figure S3.** **Parasite densities measured in 3D7 *P. falciparum* parasites during the egress assay**. Previous cycle – parasites had grown for a full cycle (48 hours) in presence **(DPG MACS and DPG Normal)** or absence **(Untreated MACS and Untreated Normal)** of 2,3-DPG 8mM; Following cycle – Schizonts recovered through magnetic separation **(DPG MACS and Untreated MACS)** and parasites kept under normal conditions (**DPG Normal and Untreated Normal**) were allowed to grow in untreated new RBCs. This graph was chosen as representative of three independent experiments performed in triplicate. (DPG MACS – 0h Mean= 1% SD=0%, 1.5h Mean= 0.51% SD=0.05%, 3h Mean=0.38% SD=0.02%, 24h Mean=0.53% SD=0.01%; DPG Normal – 0h Mean=1.31% SD=0.04%, 1.5h Mean=1.22% SD=0.03%, 3h Mean=1.40% SD=0.03%, 24h Mean=1.53% SD=0.02%; Untreated MACS – 0h Mean=1% SD=0%, 1.5h Mean=0.57% SD=0.01%, 3h Mean=0.70% SD=0.0.08%, 24h Mean=0.82% SD=0.07%; Untreated Normal – 0h Mean=7.26% SD=0.14%, 1.5h Mean=7.00% SD=0.09%, 3h Mean=8.73% SD=0.08%, 24h Mean= 8.85% SD=0.09%).

**Figure S4.** **Principal Component Analysis (PCA) with all samples + QCpools**.

**Figure S5.** **Venn-Diagram with the most relevant metabolites of the PLS-DA models** comparing infected-treated and non-infected samples versus infected untreated samples (in blue) and non-infected samples versus infected untreated samples (in black). The *m/z* values of the metabolites that can be considered as specific of infected treated samples are shown in the table.
