# Supplementary figures and images for: Synthetic Red Blood Cell-Specific Glycolytic Intermediate 2,3-Diphosphoglycerate (2,3-DPG) Inhibits Plasmodium falciparum Development In Vitro
Source: Front Cell Infect Microbiol. 2022 Mar 15;12:840968. doi: 10.3389/fcimb.2022.840968 (PMC8967366; doi:10.3389/fcimb.2022.840968)

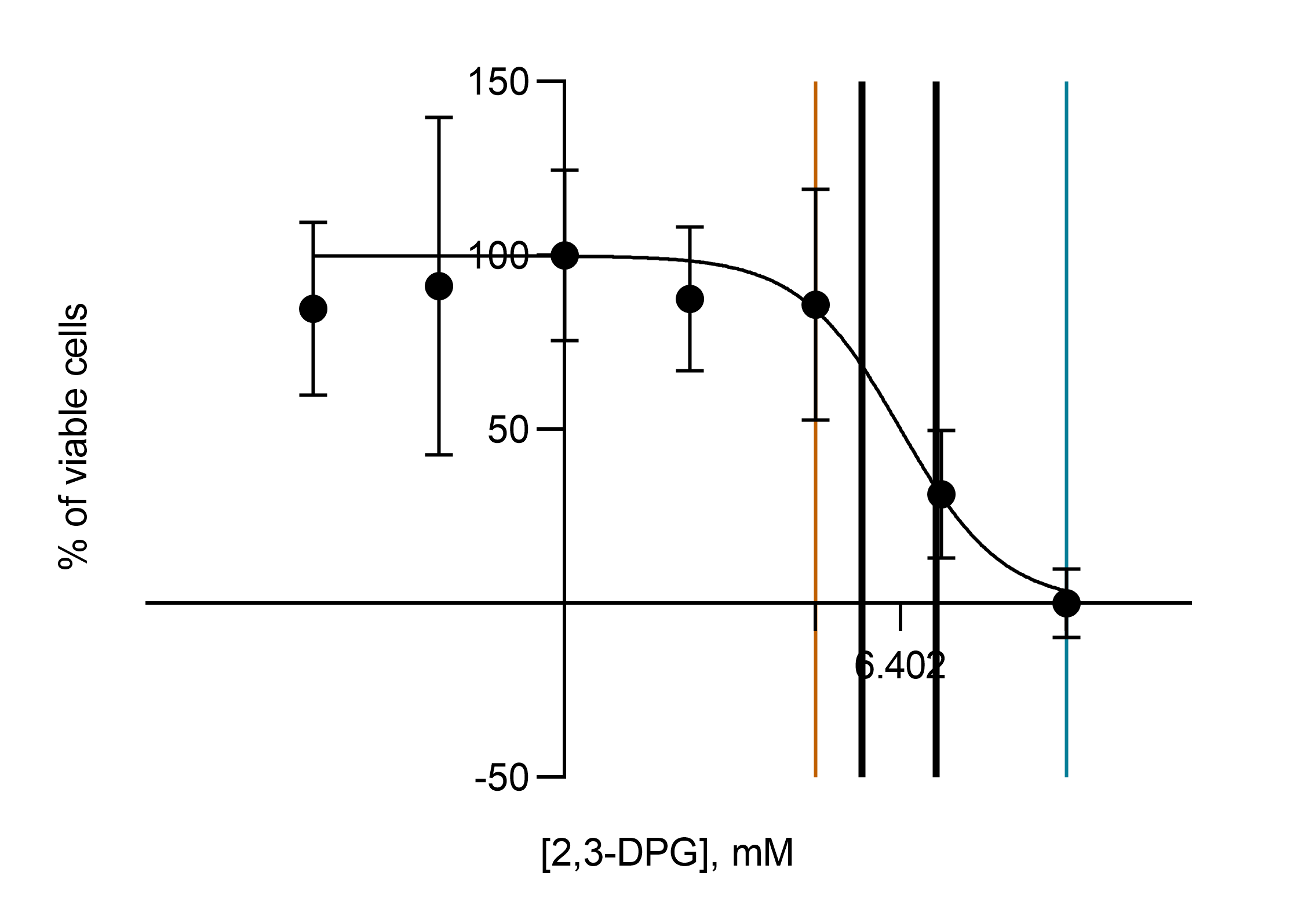

Supplement: Supplementary file 1 [file DataSheet_1.zip › suplementary material/Morais et al Figure S1.tif]

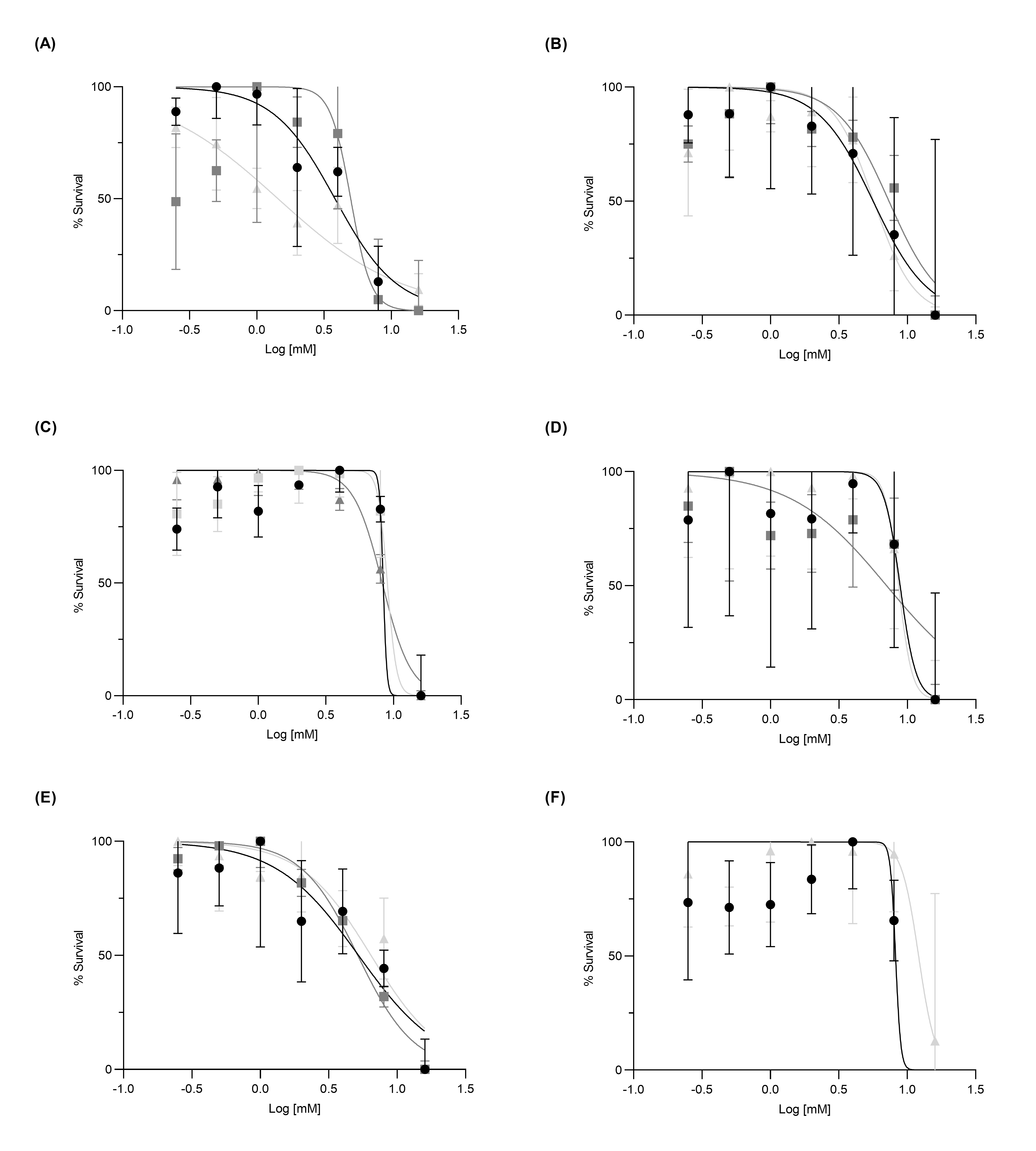

Supplement: Supplementary file 1 [file DataSheet_1.zip › suplementary material/Morais et al Figure S2.tif]

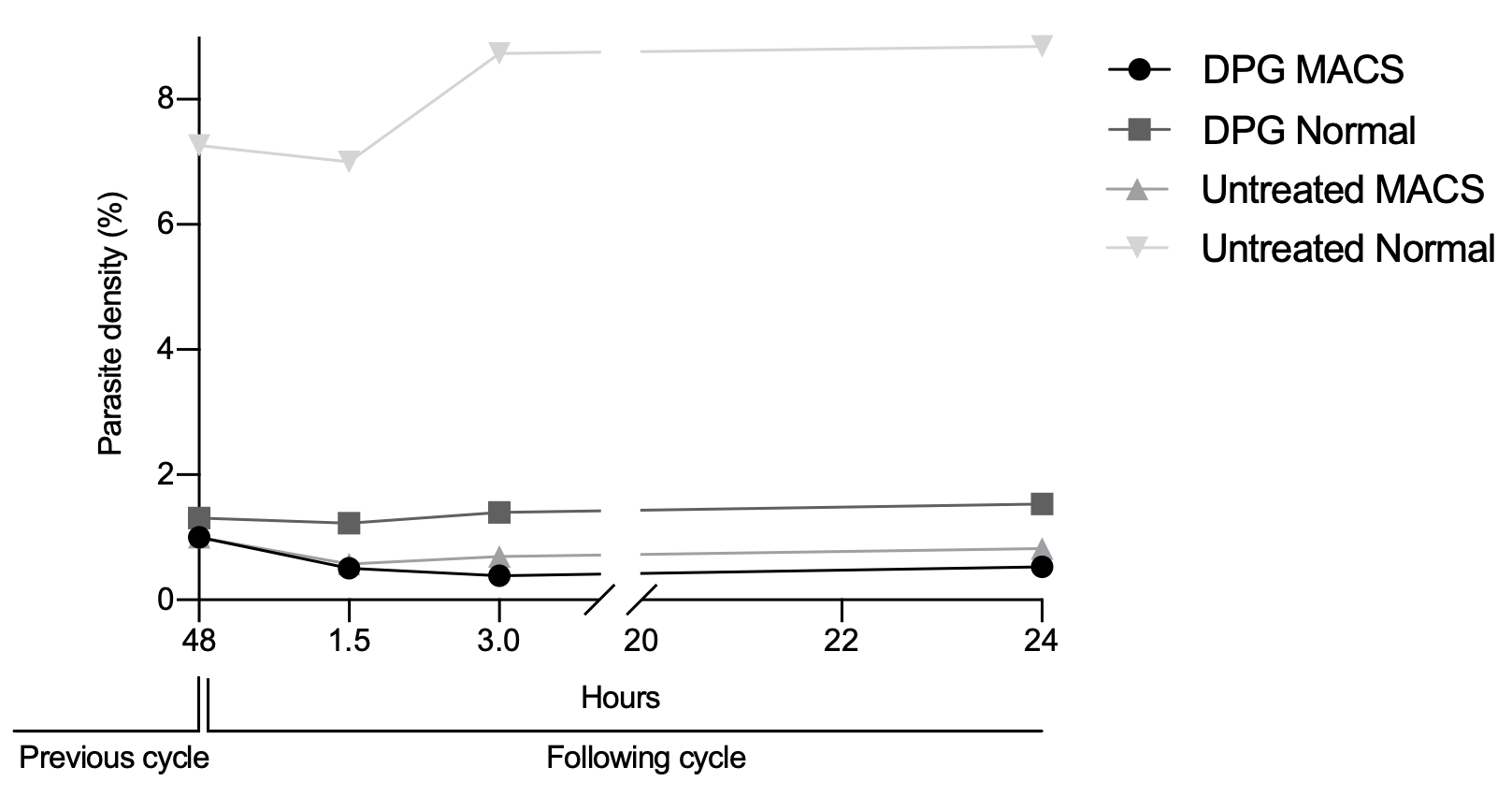

Supplement: Supplementary file 1 [file DataSheet_1.zip › suplementary material/Morais et al Figure S3.tiff]

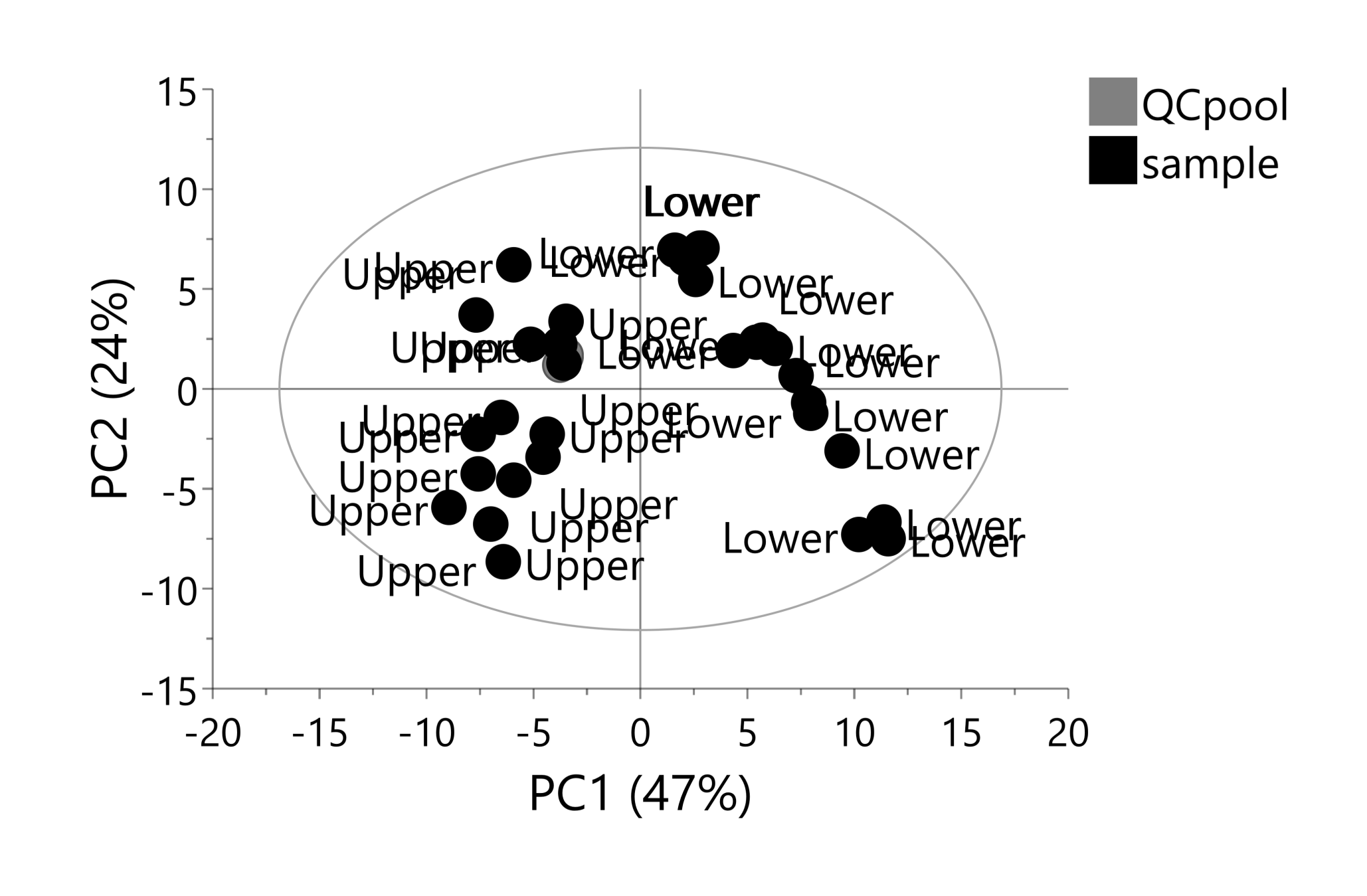

Supplement: Supplementary file 1 [file DataSheet_1.zip › suplementary material/Morais et al Figure S4.tif]

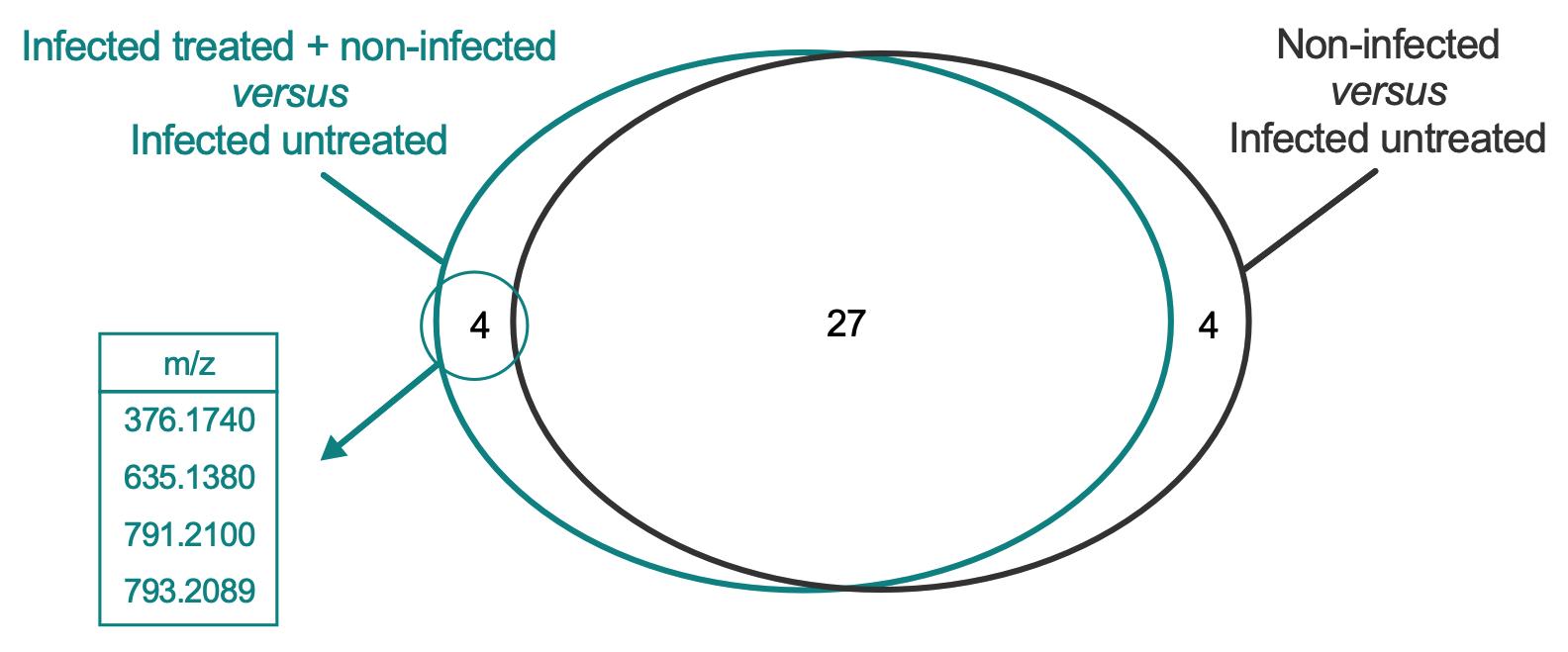

Supplement: Supplementary file 1 [file DataSheet_1.zip › suplementary material/Morais et al Figure S5.tiff]
